# Mitochondrial Morphology and Mitophagy in Heart Diseases: Qualitative and Quantitative Analyses Using Transmission Electron Microscopy

**DOI:** 10.3389/fragi.2021.670267

**Published:** 2021-05-06

**Authors:** Helen E. Collins, Mariame Selma Kane, Silvio H. Litovsky, Victor M. Darley-Usmar, Martin E. Young, John C. Chatham, Jianhua Zhang

**Affiliations:** ^1^Division of Environmental Medicine, Department of Medicine, University of Louisville, KY, United States; ^2^Division of Molecular and Cellular Pathology, Department of Pathology, University of Alabama at Birmingham, Birmingham, AL, United States; ^3^Division of Anatomic Pathology, Department of Pathology, University of Alabama at Birmingham, Birmingham, AL, United States; ^4^Division of Cardiovascular Disease, Department of Medicine, University of Alabama at Birmingham, Birmingham, AL, United States

**Keywords:** transmission electron microscopy, heart, heart failure, myocardial infarction, mitochondria, autophagy, mitophagy, aging

## Abstract

Transmission electron microscopy (TEM) has long been an important technique, capable of high degree resolution and visualization of subcellular structures and organization. Over the last 20 years, TEM has gained popularity in the cardiovascular field to visualize changes at the nanometer scale in cardiac ultrastructure during cardiovascular development, aging, and a broad range of pathologies. Recently, the cardiovascular TEM enabled the studying of several signaling processes impacting mitochondrial function, such as mitochondrial fission/fusion, autophagy, mitophagy, lysosomal degradation, and lipophagy. The goals of this review are to provide an overview of the current usage of TEM to study cardiac ultrastructural changes; to understand how TEM aided the visualization of mitochondria, autophagy, and mitophagy under normal and cardiovascular disease conditions; and to discuss the overall advantages and disadvantages of TEM and potential future capabilities and advancements in the field.

## Introduction

**Figure 1:**
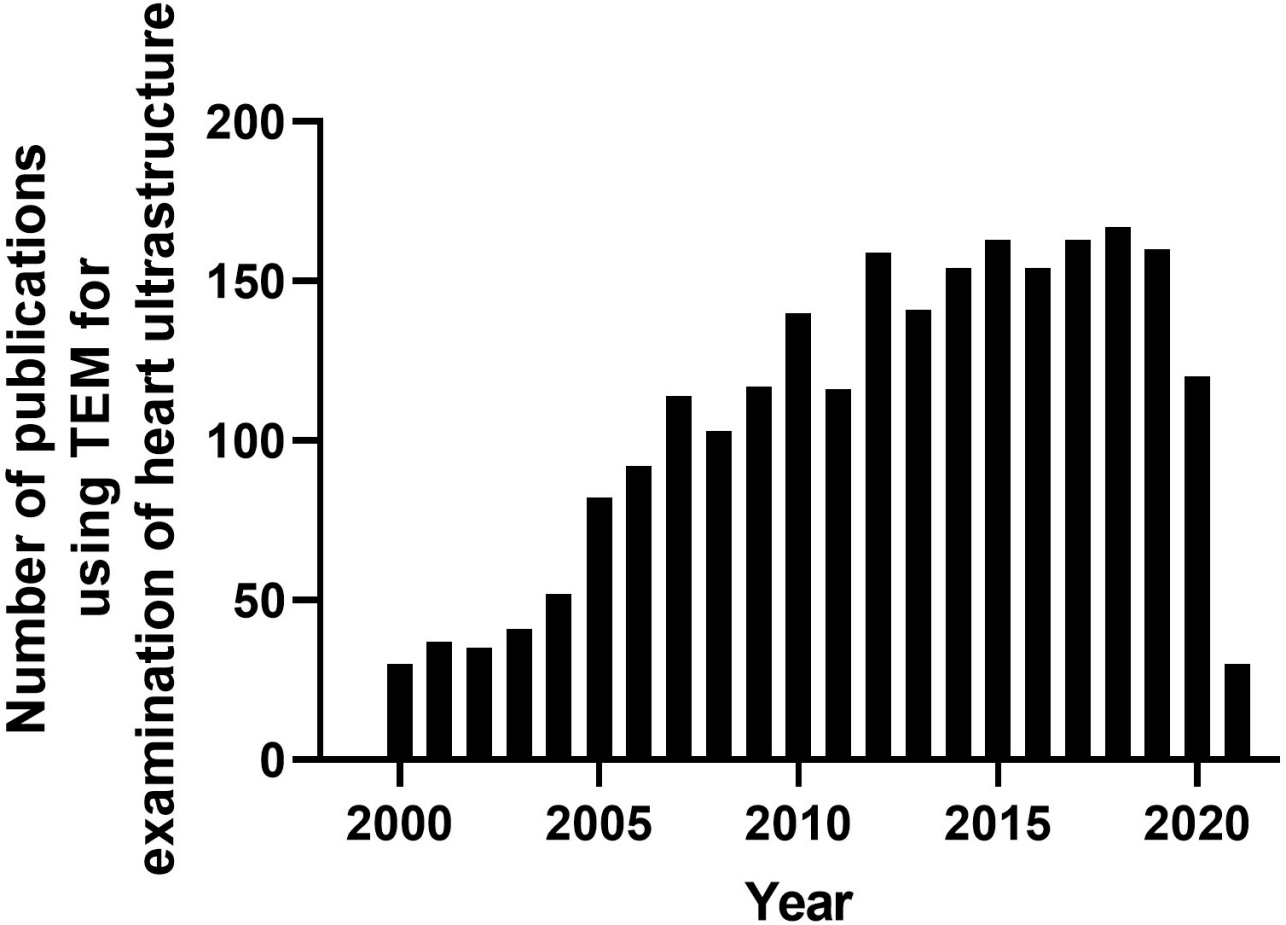
Timeline of peer-reviewed cardiovascular publications utilizing transmission electron microscopy to examine cardiac ultrastructure. Data taken from Pubmed analytics using a search of “Transmission electron microscopy” and “heart.” Data presented were taken from 2000-present.

Since the development of the first electron microscope in the 1930's, transmission electron microscopy (TEM) has been an essential technique for the visualization of cellular ultrastructure. The timeline of the key events in the development and evolution of TEM has been described in an previous study (Lukasz Mielanczyk et al., 2015). TEM has steadily gained popularity in the cardiovascular research field over the last 20 years, being utilized in over 2,000 peer-reviewed cardiovascular research publications (Figure 1), to examine cardiac ultracellular organization at the nanometer scale. In the cardiovascular field, TEM is often used to assist in studies investigating: (1) cardiovascular development and aging; (2) cardiovascular disease processes, such as heart failure, myocardial infarction/ischemia (MI), and diabetic cardiomyopathy; (3) phenotyping of cardiovascular transgenic models; and (4) examination of complex signaling processes, many of which impact mitochondrial function and dynamics, such as apoptosis, autophagy, and mitophagy. In this review, we discuss the application of TEM as a tool for viewing cardiac ultrastructure and how this has been leveraged in cardiovascular studies to visualize complex signaling processes, with a special focus on the mitochondria. Although it is important to appreciate that, in addition to TEM, scanning electron microscopy (SEM) has also been used in several cardiovascular studies, its primary application is to provide information on cell surface and composition, rather than cellular ultrastructure; therefore, SEM will not be discussed in this review (Klang et al., 2012; Lukasz Mielanczyk et al., 2015).

### Cardiac Structures Visualized With TEM

**Figure 2:**
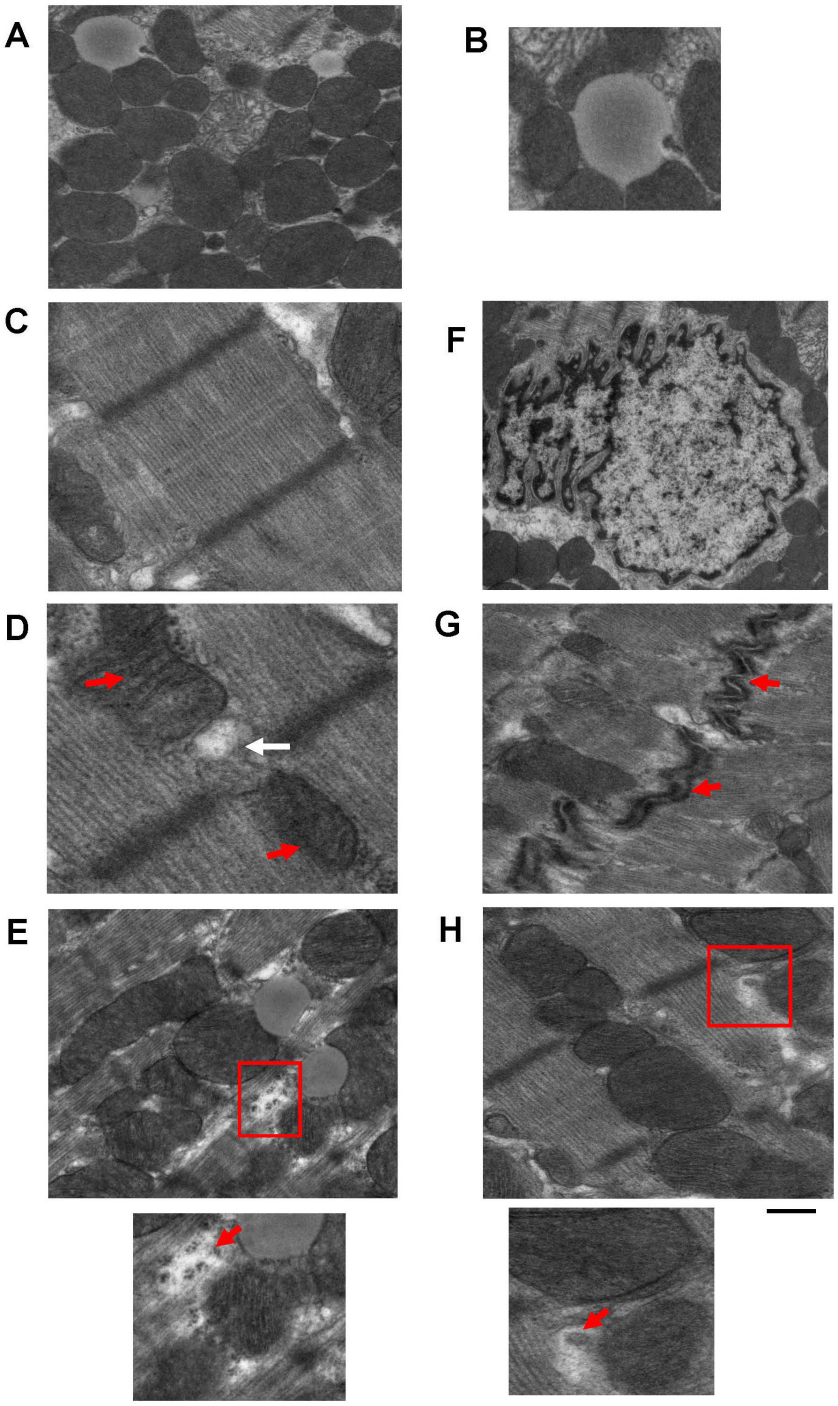
Example images of cardiac cellular structures examined using TEM studies from wild-type mouse hearts. **(A)** TEM image showing mitochondria surrounding a lipid droplet; **(B)** Magnification of Lipid droplet in **(A)**; **(C)** TEM image of cardiac sarcomeric structure; **(D)** TEM image of SR-mitochondria connections. Red arrows depict mitochondria and white arrow depicts SR; **(E)** TEM image showing glycogen deposits (highlighted in red box); **(F)** TEM image of a nucleus; **(G)** TEM image of gap junctions (gap junctions depicted by red arrows); and **(H)** TEM image showing a mitochondrial derived vesicle budding from larger mitochondria (highlighted by red box). Images are unpublished images from Zhang lab studies. Scale bar depicts 500 nm.

Transmission electron microscopy has been used to provide high magnification and high-resolution images of sarcomeric structures (i.e., H-zone, A-band, M-line, and Z-line of the sarcomere, myofibrils, etc.), and at the subcellular level, numerous structures have been characterized, such as endoplasmic/sarcoplasmic reticulum (ER/SR), nuclei and chromatin structure/organization, lipid droplets (LDs), glycogen deposits, lysosomes, autophagolysosomes, gap junctions, caveolae, and extracellular matrix (ECM), and mitochondrial-associated structures, which include cristae, intact and disrupted mitochondria, mitochondrial-derived vesicles (MDV) (Cadete et al., 2016), mitochondrial-associated membranes (MAMs), and mitophagosomes. TEM has also been used to reveal or confirm the presence of new cell populations in the heart, such as telocytes (Popescu et al., 2010; Fertig et al., 2014; Tay et al., 2017). Figure 2 shows examples of cardiac structures visualized using TEM. From the images, quantification can be performed (Glancy, 2020) for the assessment of number, length, aspect ratio, and areas of mitochondria, lysosomes, and autophagosomes, in addition to qualitatively scoring cellular integrity (e.g., cristae of mitochondria, cristae junctions, and matrix remodeling).

### TEM Sample Preparation

To obtain clear, reliable images suitable for accurate measurements, particular care must be taken at the sample preparation stage to avoid the production of artifacts. Typically, freshly isolated cardiac samples, usually left ventricular (LV) samples, are extracted and then either rinsed quickly in cold phosphate-buffered saline (PBS) or retrogradely perfused with cold PBS containing potassium chloride to arrest the heart in diastole and to remove any remnant red blood cells. Adequate removal of red blood cells prior to fixation is essential to improve fixation and subsequent image quality. Following the removal of red blood cells, tissues undergo either submersion fixation or perfusion fixation in a glutaraldehyde–paraformaldehyde containing cacodylate buffer. Most commonly, the submersion fixation is preferred for LV samples as only a small fraction of the heart is needed for TEM analysis, which allows the remaining LV tissues to be used for other downstream assays that are not compatible or optimal with the fixed tissues. For some tissues, such as those collected from humans during left ventricular assist device (LVAD) surgeries, perfusion is not possible. Prior to the submersion fixation, the heart must be cut into smaller pieces to allow for optimal fixation, and for the mouse heart, we immersed LV tissue pieces of ~1 mm^3^ in size in 1 ml of glutaraldehyde–paraformaldehyde containing cacodylate buffer. After this, a post-fixation step with osmium tetroxide and uranyl acetate containing cacodylate buffer was performed to preserve lipid structures. This post-fixation stage, although important for creating the appropriate contrast required for high-quality TEM, if prolonged (i.e., longer than 90 min), can result in protein and lipid damage and distortion of the cellular ultrastructure. Following the post-fixation, samples underwent a solvent-based dehydration step followed by embedding in epoxy resin (e.g., Epon), prior to ultra-fine sectioning and post-staining with heavy metal stains. The ultra-fine sectioning is performed using an ultramicrotome, where sections of 1 μm are cut and stained with toluidine blue. The samples are then cut further using a diamond knife so that sections of 50–70 nm are achieved, and these are placed onto a metal grid. The heavy metal staining is performed with lead citrate, which interacts with the proteins and glycogens in the tissue sample and is required to enhance image contrast.

**Figure 3:**
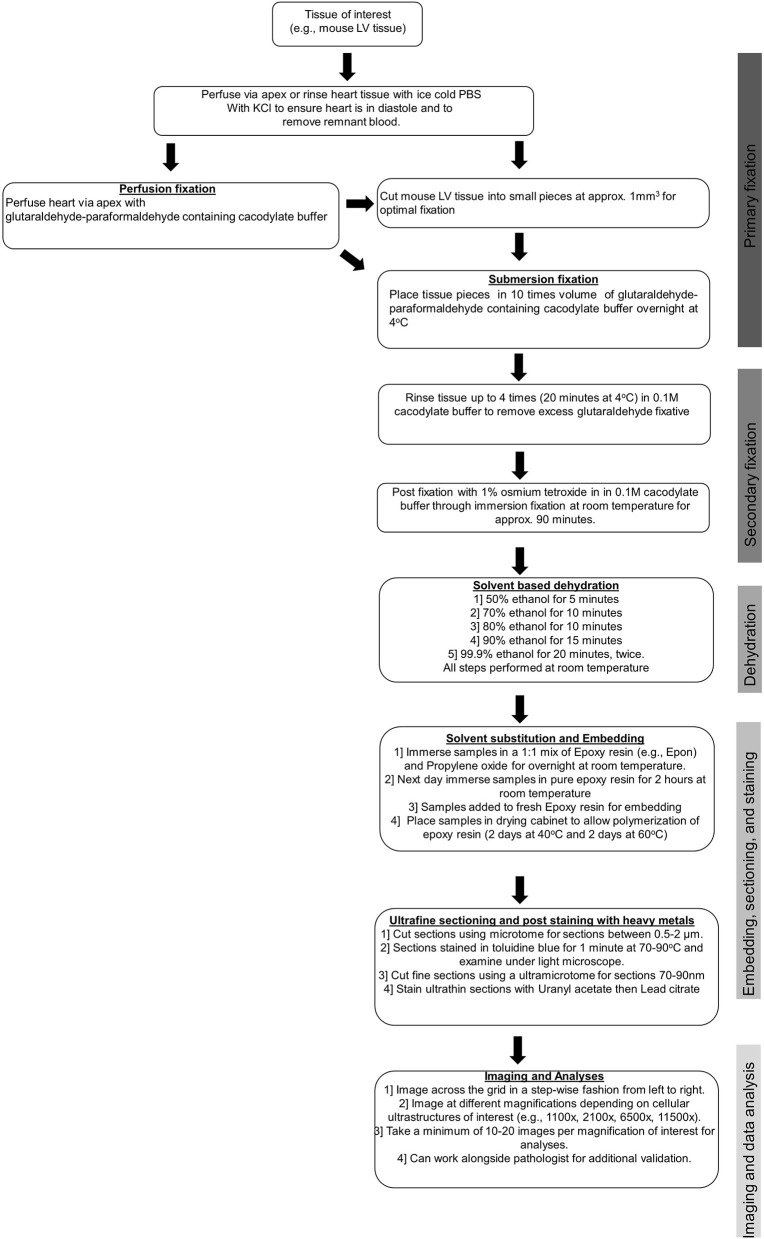
Tissue preparation for TEM studies.

Sample preparation is the single most important stage in TEM. Sample artifacts have been reported to arise from each stage in the sample preparation stage (i.e., from fixation to post-staining), which reduce resolution and can produce spurious findings. These sample artifacts include chemical fixation-induced sample contamination; small darkened speckled areas from inadequate washing; formation of precipitates by the heavy metals, uranyl acetate, and lead citrate; shrinkage artifacts introduced through rapid dehydration; tears and holes formed due to inadequate resin infiltration; sectioning of tissue resulting in compression, scratches, and tears; and high TEM beam setting resulting in tissue breakage. Some common technical problems experienced during TEM and strategies for avoiding these issues are outlined in Table 1. Therefore, with TEM, there is a constant need and requirement to improve sample preparation and the staining methods to improve image quality. Furthermore, there is a great need to adapt TEM to create innovative new methods for sample examination and quantification, which includes the use and development of immunogold labeling techniques and cryo-TEM, which have proven to be essential tools in the visualization of physiological processes and various cardiovascular pathologies (see below). Key steps of sample preparation are illustrated in Figure 3.

**Table 1:** Common technical problems with TEM and strategies for improvement.

**Technical problem**	**Impact on image quality or analysis**	**Strategies for improvement**
Under fixation of tissue	Tissue distortion and poor-quality staining	Make sure tissue is correct dimensions to allow for appropriate infiltration of fixative. Ensure appropriate volume to sample ratio. Fix tissues rapidly.
Over fixation of tissue	Tissue distortion and poor-quality staining Tissue will become brittle and hard to process	Reduce time that tissue is in fixative. Be sure that tissue is stored at appropriate temperature
Chemical fixation-induced sample contamination	Poor-quality Images	Use freshly made fixative solutions. Store fixatives correctly. Use caution and good laboratory techniques when using to avoid introduction of contaminants.
Inadequate washing	Speckled areas on tissue	Clean bottles used to store Osmium tetroxide with H_2_O_2_ and add some H_2_O_2_ to help prevent reduction of osmium tetroxide by glutaraldehyde.
Formation of precipitates (due to uranyl acetate and lead citrate)	Small black dots and speckled areas on tissue	Check pH of solutions. The pH of the uranyl acetate containing solution may need to be adjusted. Do not breathe on grids. Dry grids thoroughly. Limit light exposure for Uranyl acetate.
Inadequate resin filtration	Tears and holes in tissue	Make sure to perform solvent substitution. Resin needs to be mixed well with no bubbles. Allow appropriate time for epoxy resin infiltration and polymerization.
Toluidine blue staining	Too dark or too light	Too dark—rinse with water Too light—stain with toluidine blue longer
Imaging sections with high beam intensity	Tissue will break and curl up leaving the grid unusable	Set beam at lower intensities and slowly increase when necessary.
Image artifacts mistaken for real changes	Will lead to generation of inaccurate analyses and findings.	Learn and know how to identify common TEM artifacts. Work alongside a trained pathologist.
Analyses performed on a small number of images	May not reflect an accurate representation of what is occurring in specific tissue sample.	Take a minimum of 10–20 images per magnification of interest for analysis
Using both longitudinal and cross-sectional images for analyses	May not reflect an accurate representation of what is occurring in specific tissue sample.	Be sure to take images in longitudinal plane (unless looking at fiber changes) for analyses. Tissue pieces subjected to TEM must be representative of the sample as a whole.

### Analysis of TEM Images

Analysis of TEM images is an important step in understanding and assessing the ultrastructural changes occurring in a given tissue sample. Identification of image artifacts is a key step in the analysis of TEM images, as, to the untrained eye, these may appear to be physiological changes. The recruitment of a trained pathologist to help assess ultrastructural changes is recommended. In addition, for the quantitative analysis, it is important that at least 10–20 images are taken from each sample at each magnification and in the same plane for an accurate representation. Similarly, the tissue pieces selected for TEM must be representative of the sample as a whole to ensure accurate analysis of findings. As mentioned in Table 2, many cardiac ultrastructures have been identified, examined, and measured using TEM. For these cellular structures, descriptive analyses have often been performed to describe visual differences between samples. In addition, across the field there does not appear to be consistent reporting of TEM findings and analyses. Often, the quantitative information on cell number and size is collected during TEM analyses using software with specific cell counting and cell size capabilities, such as Image J software (National Institutes of Health, Bethesda, MD, USA). For these analyses, it is important, in terms of quantification, that cell borders are intact and completely visible in order to be included in the analyses, and care must be taken to keep measurement criteria standard across all samples in a study. In addition, it is important to normalize data obtained back to the known dimensions/area of the field assessed. Although measurements of cell size and aspect ratio (length:width) can be obtained, one significant disadvantage of TEM in terms of measuring size changes is that it does not provide a 3D image of various structures, such as mitochondria, which are spherical/tubular in form, so only crude measurements of size are possible with traditional TEM, and additional electron tomography studies are required for the assessment of 3D structures with TEM. The next section discusses the current usage and application of TEM to visualize changes in cardiac ultrastructure.

**Table 2:** Cardiac ultrastructures identified, examined, and measured using TEM.

**Cellular structures identified**	**Measurements taken**	**References**
Mitochondria	Number, size, area, aspect ratio, and contact sites with SR	Searls et al., 2004; Arany et al., 2005; Gupta et al., 2010; Ong et al., 2010; Haemmerle et al., 2011; Chen L. et al., 2012; Piquereau et al., 2012; Ahuja et al., 2013; Kubli et al., 2013; Wang et al., 2013; Jahng et al., 2015; Saito et al., 2015; Song et al., 2015a; Treskatsch et al., 2015; Eisenberg et al., 2016; Goh et al., 2016; Hall et al., 2016; Shirakabe et al., 2016; Wu et al., 2016; Beikoghli Kalkhoran et al., 2017; Takemura et al., 2017; Benador et al., 2018; Kang et al., 2018; Li et al., 2018; Sibouakaz et al., 2018; Yuan and Pan, 2018; Chaanine, 2019; Collins et al., 2019; Hsieh et al., 2019; Lampert et al., 2019; Xiong et al., 2019; Zhou et al., 2019; Babaei et al., 2020; Liang et al., 2020
Mitochondrial-derived vesicles (MDV)	Presence, distribution, and size	Cadete et al., 2016
Sarcomeres/myofibrillar structure	Structural integrity and sarcomere length	Searls et al., 2004; Gupta et al., 2010; Saito et al., 2015; Treskatsch et al., 2015; Eisenberg et al., 2016; Takemura et al., 2017; Sibouakaz et al., 2018; Yin et al., 2018; Yuan and Pan, 2018; Babaei et al., 2020
SR	Structural integrity and proximity to mitochondria	Eisenberg et al., 2016; Beikoghli Kalkhoran et al., 2017
Myelin bodies/endosomal bodies/apoptotic bodies	Presence	Gupta et al., 2010; Chaanine, 2019; Gil-Cayuela et al., 2019
Nuclei/nuclear envelope	Structure, size, and chromatin condensation	Searls et al., 2004; Gupta et al., 2010; Treskatsch et al., 2015; Chaanine, 2019
Autophagosomes/autophagic vacuoles/mitophagosomes	Number, size, and distribution	Wohlgemuth et al., 2007; Carreira et al., 2010; Ong et al., 2010; Kanamori et al., 2011a,b, 2013; Pan et al., 2012; Zhao et al., 2012; Kubli et al., 2013; Backues et al., 2014; Dupont et al., 2014; Kageyama et al., 2014; Shpilka and Elazar, 2015; Shpilka et al., 2015; Shirakabe et al., 2016; Wu et al., 2016; Takemura et al., 2017; Kang et al., 2018; Li et al., 2018; Yin et al., 2018; Chaanine, 2019; Gil-Cayuela et al., 2019; Hsieh et al., 2019; Xiong et al., 2019; Zhou et al., 2019
Lysosomes	Number, size, distribution	Carreira et al., 2010; Kanamori et al., 2011a,b, 2013; Zhao et al., 2012; Jahng et al., 2015; Gil-Cayuela et al., 2019; Hsieh et al., 2019
Lipid droplets	Number, size, proximity to autophagosomes and mitochondria	Searls et al., 2004; Caspi et al., 2013; Sibouakaz et al., 2018; Hsieh et al., 2019; Tong et al., 2019
Glycogen granules	Presence, distribution, size, and aggregation	Haemmerle et al., 2011; Saito et al., 2015; Takemura et al., 2017
Gap junctions/desmosomes	Presence and structural integrity	Hesketh et al., 2010; Caspi et al., 2013; Bonda et al., 2016; Sibouakaz et al., 2018
Telocytes	Presence and distribution	Gherghiceanu and Popescu, 2010; Gherghiceanu et al., 2010; Popescu et al., 2010; Fertig et al., 2014; Tay et al., 2017

## Use of TEM to Study Cardiovascular Disease Processes

Cardiovascular diseases, such as hypertrophy, hypertension, myocardial infarction (MI), ischemia/reperfusion (I/R) injury, heart failure (HF), and metabolic-induced perturbations in cardiac function, such as those associated with diabetic cardiomyopathy, are all characteristically associated with significant alterations in the cardiac ultrastructure. Broadly, these ultrastructural changes, most often visualized using TEM, include myofibril and sarcomeric disorganization; damaged gap junctions; changes in nuclear organization, such as chromatin aggregation; changes in mitochondrial shape, content, and distribution; swollen, cristae-damaged mitochondria; LD deposition; glycogen aggregation; changes in the SR-mitochondrial association and SR dilatation; and the presence of autophagosomes and lysosomes. Cardiac ultrastructures identified, examined, and measured using TEM are summarized in Table 2. This section discusses the current usage and application of TEM to visualize changes in the cardiac ultrastructure during these respective disease processes.

### Non-ischemic and Non-metabolic Heart Disease

As mentioned above, TEM has been utilized to document ultrastructural changes in the heart in cases of pathological remodeling, such as hypertrophy, hypertension, cardiomyopathy, and ultimately progression to HF, in various models including human tissues, animal models, and in cultured cells (Table 3). Historically, many of the TEM studies performed in the failing heart focused significantly on sarcomeric and myofibrillar structure, density, and organization. For example, degeneration of cardiac sarcomeres has been observed in the hypertrophic cardiac tissue that was associated with significant thickening of z-bands and shortening of the sarcomere and with changes in the basement membrane and tubular structure (Maron et al., 1975a). In addition, the TEM analyses of cardiac tissues from patients with hypertrophy and valvular disease showed focal myofibril lysis, loss of thick myofilaments, and T-tubular changes, which were associated with expansion of the SR and mitochondria into these areas (Maron et al., 1975b). Furthermore, TEM of LV endomyocardial biopsies showed that myofibrillar density was lower in the hearts of patients with HFrEF than those with HFpEF and was associated with significant myofibrillar loss (van Heerebeek et al., 2006). End-stage dilated cardiomyopathic heart tissue obtained from patients undergoing heart transplant showed similar changes in myofilament and sarcomeric structures, which have been linked to changes in cytoskeletal proteins including desmin (Schaper et al., 1991; Mudhar et al., 2001).

**Table 3:** Cardiac ultrastructure in hypertrophy, heart failure (HF), and hypertension.

**Human heart hypertrophy, hypertension, and HF**	**Ultrastructural changes**	**Quantitative TEM?**	**References**
Dilated cardiomyopathic (DCM) hearts compared to ischemic (ICM) hearts	A large population of smaller **mitochondria** in dilated hearts vs. ICM hearts despite similar levels of mitochondrial dysfunction, with differential changes in mitochondrial biogenesis	Quantitative morphometric measurement of mito cellular volume density (0.3 ICM vs. 0.6 DCM μm^3^/μm^3^)	Ahuja et al., 2013
Patients with DCM (250 patients)	Ultrastructural changes in cardiac **myofilaments**, disorganized **sarcomeric** structure, **mitochondrial** and **glycogen** aggregation were shown, with myofilament changes associated with ↓ survival	% myofilament changes were scored and subdivided with focal derangement, diffuse myofilament lysis, replaced by smooth endoplasmic reticulum (ER) and replaced by large filamentous masses	Saito et al., 2015
Cardiac ultrastructure in human endocardial biopsies in patients with chronic HF due to different etiology	Lysosomal storage diseases, mitochondrial cardiomyopathy, autophagic degeneration, and doxorubicin (Dox)-induced cardiomyopathy, exhibit general ↓ of myofibrils, vacuolar degeneration, accumulation of **glycogen granules**, ↑ presence of **autophagic vacuoles**, and changes in **mitochondrial** size, shape, and number	No	Takemura et al., 2017
Human DCM	The presence of **autophagic vacuoles**, electron-dense bodies similar to **lysosomes**, endosomal structures, myelinated bodies, and multivesicular bodies, with associated change of mRNA levels of 13 autophagy-related genes	No	Gil-Cayuela et al., 2019
Induced pluripotent stem cells (IPSCs) from patients with arrhythmogenic right ventricular cardiomyopathy (ARVC)	Desmosome distortion (↑ in gap and total width), and ↑ **lipid droplet (LD)** accumulation in response to proadipogenic stimulus	Desmosomal gap width 24 ->34 nm, total desmosome width 100 - >160 nm by ARVC, more gap width the more % LD containing cardiomyocytes	Caspi et al., 2013
Phenylephrine (PE)-treated rat adult cardiac myocytes, rat LV after ascending aortic banding (HF), human HF patient LV subepicardial biopsy	Time-dependent changes (stages) occurred with **mitochondrial** cristae disruption, swelling, and membrane rupture in the presence of **autophagosomes**, significant chromatin condensation and appearance of apoptotic bodies—compared with hearts from a rat HF model, and a human patient samples HF with reduced ejection fraction (HFrEF)	% for stages A, B, B–>C, C, and D, per TEM 12k× magnified field scored for ACM control vs. PE, for rat normal vs. HF, and human normal vs. HFrEF	Chaanine, 2019
**Animal models of non-ischemic and non-metabolic heart disease**	**Ultrastructural changes**	**Quantitative TEM?**	**References**
Guinea pig HF (8 weeks after ascending aortic constriction)	**Mitochondria** were fragmented and aggregated with ↓ size and area, in comparison to sham	Av. mito length ~1 −>0.75 μm, Av. mito area ~0.7 −>0.4 μm^2^	Goh et al., 2016
Cardiomyocyte expression of myotrophin in mice led to heart hypertrophy at 4 weeks of age and HF at 36 weeks. of age	At 16–24 weeks of age, **mitochondria** became swollen, with ↓ matrix granules, at >34 weeks, with disrupted cristae. **Nuclear membrane** became distorted at 16–24 weeks, some rupture at >36 weeks. **Myofibrillar and sarcomeric** organization were disrupted at the z-line at 16–24 weeks, and worsen with appearance of myelin bodies at >36 weeks.	Scoring of changes (1–4) of mitochondria, myofibril and z-line, and cytoplasm/nucleus (0 to >45% changes initiation - progression - transition)	Gupta et al., 2010
Rats with HF induced by volume overload	**Nuclear chromatin** condensation, **myofibril** damage, **mitochondrial** swelling and cristae damage, and the presence of electron-dense bodies in the **mitochondria** associated with the initiation of apoptosis	↑ from ~10 −>~75% cardiomyocytes with apoptotic and mitochondrial changes	Treskatsch et al., 2015
Adiponectin-KO mice in response to sham or mTAB	Sham hearts contain **electron-dense lysosomes** Electron-dense **lysosomal** structures are near the **mitochondria** that had substantial cristae damage, in some cases membrane rupture after transverse aortic constriction (TAC) surgery	No	Jahng et al., 2015
Mouse TAC Cardiomyocyte-specific dynamin-related protein 1 (Drp1) heterozygous (+/–) knockout (KO) exacerbated and Tat-Beclin (TB1) attenuated phenotypes	↑ Number of **autophagosomes** containing mitochondria per total number of mitochondria (**mitophagy**) 3–7 days after TAC, ↑ **Mitochondrial** mass as deduced from the TEM images (to ~1.3) at 24 h, ↓ at 3–5 days (0.7–0.8), and ↑ again at 30 days (1.3). Drp +/– ↑ mitochondrial mass to ~1.3 both sham and TAC 7 days, TB1 ↑ mitophagy/mitochondrial number from 0 to> ~0.16	Mitophagy 0 −>0.2% (days 3, 5, and 7), Mitochondrial mass changes 1 −>1.3 (24 h) −>0.7 (3–5 days) −>1.3 (30 days) by TAC, and by Drp+/– (~1.3 at sham and 7 days), TB1 ↑ mitophagy (0 −> ~0.16)	Shirakabe et al., 2016
Rat hearts containing truncated Titin	Accumulation of **autophagic vacuoles** in the titin truncated vs. wild-type (WT) mice, mitochondrial dysfunction, ↑ p62, LC3II, and ↓ cathepsin B	Number of autophagic vacuoles/field from 1.5 -> 4.5	Zhou et al., 2019
Dox-induced cardiomyopathy in rat	Similar **mitochondrial** ultrastructural changes to HF as mitochondria were swollen and with disrupted cristae	No	Babaei et al., 2020
Cardiomyocyte deletion of STIM1 (KO) compare to WT (c)	DCM at 36 weeks of age. At 12 weeks of age, ↑ LD, ↓ **mitochondrial** length, ↑ **mitochondrial** density	Av. number of LD/4400× grid^2^ 2 (c), 3.3 (KO) Av. mito length 1150 nm (c) to 900 nm (KO) Av. number of mito/4400× grid^2^ 18 (c) to 22 (KO)	Collins et al., 2019
Rat hearts in response to angiotensin II (Ang II) and effects of simvastatin (SIM)	**Mitochondrial (mito) appearance** was **graded** on a scale of 1–5 in response to angiotensin. SIM reversed the mitochondrial changes but ↑ numbers (No.) of **LDs, autophagosomes, mitophagosomes, and lysosomes**	Graded appearance; quantified: mito length, No. of swollen mito (2–8/field), mito with vacuolization, LD, autophagosome, mitophagosome, and lysosome per field	Hsieh et al., 2019
Cathepsin S KO mice and/or Ang II administration	↑ **Autophagosome** in the macrophages in the heart by Ang II, ↑ **Autophagosome** in Cathepsin S KO heart ↑↑ **Autophagosome** by Ang II in Cathepsin S KO, associated with more inflammatory cytokines and fibrosis	No	Pan et al., 2012

*Highlighted in bold are the subcellular structures observed by transmission electron microscopy (TEM)*.

Since mitochondrial dysfunction is a common feature of adverse remodeling and HF, it is not surprising that mitochondrial derangements are among the most common ultrastructural finding in TEM studies. For example, TEM revealed a large population of smaller mitochondria in dilated cardiomyopathic hearts compared with ischemic cardiomyopathic (ICM) hearts, and this was associated with differential changes in mitochondrial biogenesis (Ahuja et al., 2013). In this study, a morphometric measurement was performed to demonstrate that the mitochondrial cellular volume density is 0.3 μm^3^/μm^3^ in ICM vs. ~0.6 in the dilated cardiomyopathic heart (Ahuja et al., 2013). Furthermore, in a study involving 250 patients, ultrastructural changes in cardiac myofilaments, disorganized sarcomeric structure, and mitochondrial and glycogen aggregation were shown with TEM in patients with dilated cardiomyopathy (DCM), with myofilament changes (% changes scored) associated with reduced survival (Saito et al., 2015). A more recent study compared cardiac ultrastructure in endocardial biopsies in patients with chronic HF, lysosomal storage diseases, mitochondrial cardiomyopathy, and doxorubicin (Dox)-induced cardiomyopathy. Dependent on disease type and stage, loss of myofibrils, vacuolar degeneration, accumulation of glycogen granules, the increased presence of autophagic vacuoles and changes in mitochondrial size, shape, and number have been observed. The importance of TEM as a tool in differential cardiac disease diagnoses was highlighted by the recommendation that tissues from every endocardial biopsy should be saved for the cardiac TEM analysis (Takemura et al., 2017). The presence of autophagic structures in the heart tissue has also been observed using TEM in cases of human DCM (Te Rijdt et al., 2016; Gil-Cayuela et al., 2019). These included autophagic vacuoles, electron-dense bodies similar to lysosomes, endosomal structures, myelinated bodies, aggresomes, and multivesicular bodies (Gil-Cayuela et al., 2019). In this same study, RNA-seq analysis showed that, out of 175 autophagy-related genes detected, these ultrastructural changes were associated with five increased and eight decreased transcripts (Gil-Cayuela et al., 2019). Analysis of autopsied and explanted hearts from patients with DCM and with phospholamban mutations showed the presence of p62 and LC3 containing aggresomes at TEM, suggestive of changes in autophagy (Te Rijdt et al., 2016).

In addition, TEM has been used to examine changes in cell culture models of cardiomyopathy. For example, induced pluripotent stem cells (IPSCs) from patients with arrhythmogenic right ventricular cardiomyopathy (ARVC) were shown to have distortion of the cell-to-cell adhesion cell type, desmosome (desmosomal gap width and total desmosome width scored), and extensive LD accumulation (scored as % LD containing cardiomyocytes), which were further augmented in response to proadipogenic stimulus (Caspi et al., 2013). In a study comparing phenylephrine (PE)-treated adult cardiomyocytes, hearts from a rat transverse aortic constriction (TAC) model and from human patients with systolic HF, it was reported that similar time-dependent changes (scored as stages A–D) occurred with mitochondrial cristae disruption, swelling, and membrane rupture in the presence of autophagosomes, in the condensation of significant chromatins, and in the appearance of apoptotic bodies (Chaanine, 2019).

Dilated cardiomyopathy is commonly associated with truncations in the large structural protein, titin. TEM studies in rat hearts containing truncated titin showed the presence of damaged mitochondria and the accumulation of autophagic vacuoles (with numbers scored per field) vs. wild-type (WT) hearts, suggestive of changes in autophagy. These changes were associated with hyperacetylation of mitochondrial proteins, increased p62 and LC3II, and decreased cathepsin B (Zhou et al., 2019). Dox-induced cardiomyopathy was shown to have similar mitochondrial ultrastructural changes to HF as mitochondria were swollen and exhibited disruption of cristae (Babaei et al., 2020). However, WT mice subjected to TAC or sham were shown to have a normal mitochondrial morphology and sarcomeric structure and organization. Since adiponectin plays a key role in DCM, adiponectin-KO mice were examined in the same study. Compared to WT, cardiac tissues in sham adiponectin-KO mice contained electron-dense lysosomes, and following TAC, these electron-dense lysosomes were located near the mitochondria that have substantial cristae damage and in some cases membrane rupture (Jahng et al., 2015). Collectively, these studies have used TEM to highlight significant structural changes in mitochondria and autophagic processes in DCM.

Many of the cardiac ultrastructural changes documented in human cardiac samples have also been examined and visualized in animal cardiac samples through the use of TEM. For example, in a guinea pig model of HF induced by pressure overload, mitochondria were fragmented and aggregated with decreased size and area, in comparison to mitochondria in sham hearts. Quantitative changes were reported for mitochondrial diameters and areas with TEM images (Goh et al., 2016). In an extensive TEM-based ultrastructural study of the transition from hypertrophy to HF in a myotrophin overexpression (OE) mouse model, mitochondria were found to become swollen and cristae were found to be disrupted. In addition, the nuclear membranes were distorted, and the myofibrillar and sarcomeric organization was disrupted at the z-line and was associated with the presence of myelin bodies, immediately prior to and during the progression to HF phase (Gupta et al., 2010). In this study, mitochondrial changes were scored as matrix electron lucent appearance, swelling, and concentric cristae; myofibril structure and z-line changes were scored as wavy, blurring, and no defined structure; and cytoplasm and nucleus were scored according to the shape change, nuclear membrane distortion, and rupture of cell membrane (Gupta et al., 2010). Rats with HF induced by volume overload were shown to exhibit changes, such as nuclear chromatin condensation, myofibril damage, mitochondrial swelling, and cristae damage, and the presence of electron-dense bodies in the mitochondria associated with the initiation of apoptosis (Treskatsch et al., 2015). In this study, the percent of cardiomyocytes with apoptotic and mitochondrial changes were scored (Treskatsch et al., 2015). In a study in C57BL/6J mice subjected to TAC, mitophagy was found to be upregulated as early as 3 days after pressure overload, and mitochondrial mass was deduced from the TEM images to be up at 24 h and down at 3–5 days after pressure overload and up again at 30 days (Shirakabe et al., 2016). The number of autophagosomes containing mitochondria per total number of mitochondria (mitophagy) was scored with the mitochondrial mass normalized to control (Shirakabe et al., 2016). In the same study, cardiomyocyte-specific heterozygous knockout (KO) of dynamin-related protein 1 (Drp1) resulted in a greater mitochondrial mass at 7 days and exacerbated HF phenotypes. Injection of Tat-Beclin 1, an inducer of autophagy, partially rescued the phenotypes (Shirakabe et al., 2016). In addition, cardiomyocyte-specific KO of STIM1, which is a key calcium signaling protein involved in cardiac hypertrophy, developed DCM at 36 weeks of age, which was associated with decreased mitochondrial length, an increase in mitochondrial density, and an increase in the number of LDs from 12 weeks of age (Collins et al., 2019).

Extensive TEM analyses were performed in hypertrophic rat hearts to investigate the potential cardiac benefits of the 3-hydroxy-3-methyl-glutaryl-coenzyme A (HMG-CoA) reductase inhibitor, simvastatin (SIM), in response to 14 days of angiotensin II (Ang II) osmotic pump infusion (Hsieh et al., 2019). In this study, the mitochondrial appearance was subjectively graded in control, Ang II, and Ang II + SIM on a scale of 1–5 with 1 referring to normal mitochondria, 2 referring to occasional swollen, 3 referring to major distortions, 4 referring to membranes/cristae dissociated into particulates, and 5 referring to mitochondria with damaged cristae, mitochondrial membrane damage, and mitochondrial vacuolization. In addition, the mitochondrial length (decreased with Ang II vs. control), the number of swollen mitochondria (increased in Ang II vs. control), mitochondrial vacuolization (increased in Ang II vs. control), LDs, autophagosomes, mitophagosomes, and lysosomes (increased in Ang II vs. control) were quantified. Interestingly, SIM reversed the Ang II-induced mitochondrial changes but increased the numbers of LDs, autophagosomes, mitophagosomes, and lysosomes in response to Ang II (Hsieh et al., 2019). Cathepsin S deficiency was shown to result in more fibrosis and more inflammatory cytokines in the heart in response to Ang II. These pathologies were also associated with more autophagic vacuoles in macrophages in the heart (Pan et al., 2012).

Collectively, these studies show that many significant ultrastructural changes occur with pressure overload and HF, which are associated with changes in mitochondrial morphology and accumulation of autophagic vacuoles. More than half of these studies performed morphometric or graded quantifications of ultrastructural changes in diseases or disease models. These observations are useful in the investigation of pathologies and effects of potential therapeutic interventions in both human patient specimen and animal models.

### Ischemic Heart Diseases

Many of the ultrastructural changes occurring with non-ischemic heart diseases also occur in I/R and MI despite the differences in the underlying pathology (Table 4). Compared to HF tissues, the ultrastructural studies in ICM are relatively fewer, and most studies lack quantification of the changes. However, TEM has been used to examine changes in cardiomyocyte apoptosis in hearts with ICM (Abbate et al., 2007). In addition, human LV tissues subjected to ischemia showed damaged contractile proteins and cytoskeletal structures, which were associated with the disruption of several cytoskeletal proteins, including desmin, tubulin, and myomesin (Hein et al., 1995).

**Table 4:** Cardiac ultrastructure in ischemia/reperfusion (I/R) and myocardial infarction (MI).

**Animal models of I/R and MI**	**Ultrastructural changes**	**Quantitative TEM?**	**References**
A rat model of MI	**Myocardial fiber** network is disrupted. **Mitochondria** became smaller and round. ↑ Mitochondrial and **autophagosome** numbers. ↑ LC3II/I ratio and ↓ Parkin protein level. ↓ autophagic flux.	No	Wu et al., 2016
A mouse model of MI	↑ Large vacuoles resemble **autophagosomes** at the border zone and smaller autophagosomes at the remote zone of the infarct at 1-week post-MI. By 3-week post-MI, more autophagosomes and lysosomes were present in the remote zone, and autophagosome size in the border zone was smaller. Rapamycin ↑ presence of autophagosomes and lysosome, whereas hearts from bafilomycin injected group remained free of these organelles.	No	Kanamori et al., 2011a
MI in GFP-LC3 transgenic mice, treated with starvation and/or bafilomycin	Time-dependent ↑ of **autophagosomes** and **electron-dense bodies** at both the infarct and border zones following MI from 30 min to 24 h. ↑ Cathepsin D at 30 min−24 h, whereas LC3II at 4–24 h and p62 at 24 h. Immune-TEM with a cathepsin D antibody and enzyme cytochemistry for acid phosphatase were used to confirm that the electron-dense bodies were in fact lysosomes. Starvation ↑ autophagosomes and bafilomycin ↑ electron-dense bodies.	No	Kanamori et al., 2011b
Post-MI treatment of resveratrol	↑ **Autophagic vacuoles and lysosomes** in post-MI hearts, which were further ↑ with resveratrol and associated with a reduction in adverse remodeling. ↑ **Autophagic vacuoles and lysosomes** by resveratrol and attenuated by adenosine monophosphate-activated protein kinase (AMPK) inhibitor compound C in neonatal cardiomyocytes *in vitro*. ↑ Electron-dense lysosomes without increase of autophagosomes by chloroquine.	No	Kanamori et al., 2013

*Highlighted in bold are the subcellular structures observed by TEM*.

In addition, there have been some studies that have used TEM to examine cardiac tissues extracted during LVAD placement and during transplantation. For example, TEM has been used to examine cardiac tissue in patients with end-stage HF before and after LVAD placement and to examine soft tissue reaction to LVAD placement. In these studies, it has been shown that there are significant ultrastructural changes that occur in the hearts of these patients, which include basal membrane changes (Bruggink et al., 2007), changes in collagen fibers and remodeling (Bruggink et al., 2007), changes in myofilament structure, and changes in mitochondrial size and number (Ikeda et al., 2015). A recent study compared pre-LVAD and post-LVAD myocardial tissue samples from patients with DCM and ischemic heart disease. It was found that, although mitochondrial morphology was similar between samples from patients with ischemic heart disease and DCM, differences were observed in some mitochondrial proteins (de Weger et al., 2011). TEM has been used to examine aortic valve ultrastructure in patients with LVAD and non-LVAD during transplant, where it has been shown that there was an increase in the fiber size in patients with LVAD, which was linked to reduced compliance and changes in proteins associated with the valve activation (Stephens et al., 2018). Of note, many of these ultrastructural changes are not impacted by LVAD placement. For example, it has been shown that after LVAD unloading, the LV structure was not impacted and was not indicative of atrophic changes (Diakos et al., 2014).

Similarly, the cardiac ultrastructural changes have been observed in animal models of ischemic heart disease. In a rat model of MI, TEM showed that the myocardial fiber network was disrupted, mitochondria were smaller and rounder, and mitochondrial and autophagosome numbers were also increased. These ultrastructural changes were associated with increased LC3II/I ratio and decreased Parkin protein level, with decreased autophagic flux as assessed by chloroquine treatment (Wu et al., 2016). In a mouse model of MI, there was an increase in large vacuoles that resembled autophagosomes at the border zone and smaller autophagosomes at the remote zone of the infarct at 1-week post-MI (Kanamori et al., 2011a). By 3-week post-MI, more autophagosomes and lysosomes were present in the remote zone, and autophagosome size in the border zone was smaller (Kanamori et al., 2011a). The time-dependent accumulation of autophagosomes and electron-dense bodies, at both the infarct and border zones following MI from 30 min to 24 h, was shown in GFP-LC3 transgenic mice (Kanamori et al., 2011b). Immune-TEM with a cathepsin D antibody and enzyme cytochemistry for acid phosphatase were used to confirm that the electron-dense bodies were, in fact, lysosomes. The immunoblot analysis reported the increase of Cathepsin D from 30 min to 24 h. This study highlighted the combined use of TEM and organelle-specific labeling method in examination of cardiac ultrastructure. The accumulation of autophagosomes was also shown with increased GFP-LC3 puncta in both border and infarct areas at 30 min and in border but not infarct areas at 4–24 h. LC3II is increased at 4–24 h, and p62 increased at 24 h. Furthermore, the impact of starvation (24 h before MI) appeared to increase autophagosomes that encircle partially degraded intracellular materials and/or Bafilomycin A treatment (30 min, 0.3 mg/kg before MI, blocking autophagy and qualitatively appeared to increase electron-dense lysosomes) on both the infarct sizes (Kanamori et al., 2011b). TEM was also used to qualitatively demonstrate increased accumulation of autophagic vacuoles and lysosomes in post-MI hearts, which were further increased with resveratrol and were associated with a reduction in adverse remodeling (Kanamori et al., 2013). The effect of resveratrol on autophagic vacuoles was recapitulated qualitatively and attenuated by the adenosine monophosphate-activated protein kinase (AMPK) inhibitor compound C in neonatal cardiomyocytes *in vitro* (Kanamori et al., 2013). Collectively, these studies have highlighted the use of TEM to examine ultrastructural changes during ICM, and these studies, like those in non-ICM, have shown extensive changes in mitochondria and autophagic signaling.

### Metabolic-Induced Cardiac Perturbations

Diabetic cardiomyopathy, obesity, and utilization of high fat diet (HFD) have been shown to impact the structure and function of the myocardium, mitochondrial function, and mitochondrial driven processes (Bournat and Brown, 2010; Duncan, 2011; Miotto et al., 2018). Hearts from both diabetic and obese patients were the first to suggest changes in lipid content and lipotoxicity in hearts. The increased presence of LDs or cardiac steatosis has been observed through the use of TEM in diabetes (Sugawara et al., 2021). TEM analyses in hearts of type 2 diabetics exhibited the presence of fragmented mitochondria (Montaigne et al., 2014); however, the study also investigated parameters regarding mitochondrial function in the heart samples from patients with obesity, TEM analyses were not performed under these conditions, and the TEM analyses in this study were performed in right atrial tissue. Similar to human studies, the TEM analyses have documented the effect of diabetes and obesity on cardiac ultrastructure in animal models (Howarth et al., 2000; Searls et al., 2004; Boudina et al., 2007; Li et al., 2020). Earlier studies using a rat model of type 1 diabetes (STZ) documented no specific changes in sarcomere lengths, myofibrils, and mitochondrial appearance in hearts at 4 and 8 months after following diabetes induction (Howarth et al., 2000). A later study examined hearts from rats with STZ-induced diabetes and observed damaged mitochondria (i.e., disrupted cristae), myofibrillar disarray, nuclear envelope changes, and increased lipid deposition (Searls et al., 2004). The changes in mitochondrial area in the cell and collagen fiber cross-section area were attenuated by exercise, whereas the area of cytoplasm and LDs were not affected (Searls et al., 2004). In addition, several studies in diabetic mice and rats showed increased cardiac numbers of LDs, increased mitochondrial size, and changes in glycogen levels (Zhou et al., 2000; Christoffersen et al., 2003; Boudina et al., 2007; Li et al., 2020). In contrast, studies in obese Zucker rats showed that there were no changes in LDs or changes in mitochondrial mass and density; despite the lack of change in LDs, cardiac TAG levels were increased (Holloway et al., 2011). However, it is important to note that the ultrastructural changes found in diabetic hearts in animal studies are dependent on the animal model used, duration of diabetes, or the model used to generate diabetes, and some dynamic changes may not be effectively captured in these studies (Table 5).

**Table 5:** Cardiac ultrastructure metabolic-induced cardiac perturbations.

**Models of diabetes and responses to high fat diet (HFD)**	**Ultrastructural changes**	**Quantitative TEM**	**References**
Rats 4 and 8 months after STZ (60 mg/kg)-induced diabetes	No changes noted for **sarcomere** lengths, basal laminar membrane thickness, papillary muscle and ventricular muscle for myofibrils, z-lines, and **mitochondrial appearances**	Sarcomere lengths quantified to be ~2 μm for both ventricle and papillary muscle at 4 or 8 months after STZ	Howarth et al., 2000
Rats 7 weeks after STZ (65 mg/kg)-induced diabetes (7 weeks) and the effect of exercise (starts 2 weeks before STZ until end of study) examined	↑ Damaged **mitochondria, myofibrillar** dysregulation, **nuclear envelope** changes, and **lipid** deposition 7 weeks after STZ	Grading: 1, fully intact; 2, <50% disruption of inner mito membranes; 3, >50% disruption of inner membranes; 4, disruption of the outer membrane only; 5, disruption of both inner and outer membranes ↓ %Mito/intracellular area from 45% to 38%, ↑ collagen fiber cross-section area 35–42 × 10^−4^ mm^2^, by sedentary diabetic but not exercised diabetic. ↑ % cytoplasmic area (14%−20% or 18%), ↑ Area of lipid droplets (LD)/μm^2^ 2%−9% by both sedentary diabetic and exercised diabetic	Searls et al., 2004
Rabbit (24 prepubertal New Zealand white) with HFD 3 months	↑ **LD**, the presence of swollen, cristae damaged **mitochondria**, disorganized **myofibril** and **sarcomeric** structure, and damaged **gap junctional** regions, in both sexes	No	Sibouakaz et al., 2018
Wild-type (WT), atg7f/f:αMHC-cre, or Parkin KO mice	HFD for 2 months produced bigger **LD** in the heart of Atg7f/f:αMHC-cre, or Parkin KO mice, compared to WT	0.3 μm in WT, 1.0 μm in Atg7f/f:αMHC-cre, and 0.7 μm in Parkin KO mice	Tong et al., 2019
Rat late exercise preconditioning (LEP), exhaustive exercise (EE), and the use of wortmannin i.p. (W)	Changes in cardiac **mitochondrial** morphology and **myofibrillar** structure, and cardiac ischemia and troponin I in plasma, have been shown with different exercise groups and with wortmannin	No	Yuan and Pan, 2018

*Highlighted in bold are the subcellular structures observed by TEM*.

High fat diet also induced significant changes in rabbit cardiac ultrastructure consisting of the accumulation of LDs, the presence of swollen, cristae damaged mitochondria, disorganized myofibril and sarcomeric structure, and damaged gap junctional regions (Sibouakaz et al., 2018). The diameters of LDs have been quantified using TEM after 2 months of HFD in the hearts of WT, Atg7, or Parkin-deficient mice (Tong et al., 2019), which indicated the presence of larger LD in hearts of both the Atg7 KO and Parkin KO mice vs. WT hearts. Furthermore, cardiac mitochondrial morphology and myofibrillar structure have been shown to be altered by different exercise protocols (Yuan and Pan, 2018). Together, these studies have shown that TEM is an important tool in assessing the contribution of diabetes and obesity with the development of lipotoxicity and similar to many of the previous cardiac studies also have shown changes in autophagy.

### Aging

Aging increases the risk for the development of cardiovascular disease and is associated with changes in sarcomeric and mitochondrial structure. TEM analyses have been performed to investigate aging-related changes in cardiac ultrastructure (Table 6). Earlier studies have demonstrated that the presence of cardiac autophagic vacuoles is increased by caloric restriction in aging rat hearts (26 months of age) (Wohlgemuth et al., 2007). Dysregulation of gap junction and intercalated disc morphology have been observed *via* TEM in aged mouse hearts (24-month-old) in comparison to hearts from younger mice (4-month-old). These changes in gap junction morphology were associated with decreased Cx43, increased β-catenin, and increased collagen (Bonda et al., 2016). In addition, a study by Eisenberg et al. showed a significant decrease in mitochondrial volume, a decrease in myofibrillar volume, and an increase in sarcoplasmic volume in aging hearts (Eisenberg et al., 2016). They also showed that spermidine feeding from 18 to 24 months of age improved cardiac diastolic function and restored these structural changes to close to those in the young (4-month-old) mice (Eisenberg et al., 2016). Notably, the spermidine cardiac protective effect is dependent on the autophagy gene *Atg5* (Eisenberg et al., 2016). However, a more recent study demonstrated the presence of elongated mitochondria of aging hearts, termed as “megamitochondria,” which was associated with the increase of mitochondrial-associated p62 and Parkin and decreased autophagy-related 9B (Atg9b), nuclear respiratory factor 1 (Nrf1), and mtDNA/nDNA (Liang et al., 2020). The differences between these two studies may be due to fact that the volumes of mitochondria were normalized to the volumes of the myocytes in the first study, and the absolute area visualized in TEM was used for the latter study. Collectively, these TEM studies show ultrastructural changes in the aging hearts that are associated with significant changes in mitochondrial morphology and autophagy.

**Table 6:** Aging-induced cardiac perturbations.

**Aging models**	**Ultrastructural changes**	**Quantitative TEM**	**References**
Male Fisher 344 rats 6 and 26 months	↑ Cardiac **autophagic vacuoles** by caloric restriction in 26-month-old rat hearts	Mean fractional autophagic vesicle volume is increased from 0.7 to 1.4 by caloric restriction in 26-month-old rat heart	Wohlgemuth et al., 2007
Male mice 4–24 months	Dysregulation of **gap junction and intercalated disc** morphology	No	Bonda et al., 2016
Male mice 4 vs. 24 months	↓ **Mitochondrial** volume/myocyte (mi/myo), **myofibrillar** volume (mf/myo), ↑ **sarcoplasm** volume (sp/myo) by age, spermidine restore these changes to nearly 4 months level	Mi/myo from 0.35 to 0.28, mf/myo from 0.53 to 0.51, sp/myo from 0.10 to 0.19 from 4 to 24 months, spermidine restore these values	Eisenberg et al., 2016
Male mice 4–24 months	Elongated **mitochondria**, “megamitochondria,” in aged mice	Mitochondrial area increased from 0.6 to 1 μm^2^	Liang et al., 2020

*Highlighted in bold font are the subcellular structures observed by TEM*.

Overall, these studies all suggest that the most common TEM observations in models of cardiac physiological and pathological remodeling are those associated with changes in mitochondrial morphology and distribution, and several studies show a link to alterations in autophagy through the visualization of autophagosomes and lysosomes. The next section of the review specifically examines the usage of TEM to visualize autophagy and mitophagy in cardiac tissue.

## Use of TEM to Visualize Changes in Autophagic Mechanisms and Pathways

A common theme in many of the studies discussed in the previous section is the contribution of substantial changes in mitochondrial form and function, and mitochondrial quality control processes, such as autophagy and mitophagy, to the dysregulation of the heart during disease. TEM has been heavily utilized for the examination of autophagy due to the ability to visualize autophagosomes and vacuolar structures (Yla-Anttila et al., 2009; Backues et al., 2014) and has been a recommended methodology (Klionsky et al., 2016) for visualizing autophagosomes. This section discusses the application of TEM to visualize changes in cardiac autophagy and mitophagy in transgenic and pharmacological animal models.

### Autophagy

Transmission electron microscopy is an essential tool for visualizing autophagy in the heart and has been described as key to the visualization and quantification of autophagosomes and lysosomes. TEM has proved useful in the assessment of ultrastructural changes in response to starvation-induced autophagy in HL-1 cells, which have been shown to be associated with a decrease of mitochondrial content and an increase in the number of autophagolysosomes (Carreira et al., 2010). As described above, several TEM studies in biopsies from human patients, rat HF, and PE-treated rat adult cardiac myocytes have revealed the presence of autophagic vacuoles (Takemura et al., 2017; Chaanine, 2019; Gil-Cayuela et al., 2019) (Table 3). The numbers of autophagic vacuoles were quantified in rat HF and hypertension models with truncated titin and exposure to Ang II and SIM (Hsieh et al., 2019; Zhou et al., 2019) and qualitatively evaluated in Ang II model in combination with cathepsin S KO (Pan et al., 2012) (Table 3). Qualitative analyses were performed in rat and mouse MI models (Kanamori et al., 2011a,b, 2013; Wu et al., 2016) (Table 4). Several studies have used TEM in combination with manipulations that promote or inhibit autophagy to examine changes in autophagy. An aging study measured autophagic vesicle volume in hearts from the 26-month-old rat with *ad libitum* vs. caloric restriction feeding (Wohlgemuth et al., 2007) (Table 6). In the mouse MI models (Table 4), 1 week of daily rapamycin injections increased the presence of autophagosomes and lysosomes, whereas bafilomycin-injected animals remained free of these organelles in the heart (Kanamori et al., 2011a). Starvation increased autophagosomes and bafilomycin given prior to MI increased electron-dense bodies in the ischemic border zone in mouse heart (Kanamori et al., 2011b). The autophagy inhibitor, chloroquine, increased the presence of lysosomes without changing autophagosomes in a neonatal cardiomyocyte MI model; in contrast, resveratrol increased autophagosomes (Kanamori et al., 2013).

Many studies have also examined cardiac ultrastructural changes in response to the genetic manipulation of autophagy genes. As mentioned above, cathepsin S deficiency was shown to result in more autophagosomal vacuoles and exacerbates the response to Ang II (Pan et al., 2012) (Table 3). Mice with cardiac-specific knockdown of *Atg7* or whole-body KO of Parkin showed the presence of large diameter LDs associated with mitochondria in the heart, which was confirmed using standard biochemical staining of LDs (i.e., Oil Red O staining) (Table 5) (Tong et al., 2019). Knockdown of the mitophagy receptors, Bcl2 interacting protein (*Bnip3*), and FUN14 domain containing 1 (*Fundc1*) were associated with the presence of “donut”-shaped mitochondria containing electron-dense areas upon differentiation of adult cardiac progenitor cells (Lampert et al., 2019) (Table 7). Knockdown of *Atg2, Atg9*, and *Atg18* genes was known to play a role in autophagosome formation, in drosophila muscle, and in heart and was found to elongate mitochondria (Xu et al., 2019). The TEM phenotypes were associated with cardiac hypertrophy and shortened life span (Xu et al., 2019). Taken together, these studies indicate that TEM has been a useful tool to visually document autophagy in the heart.

**Table 7:** Autophagy and mitophagy in the heart (not included in previous tables).

**The models**	**Ultrastructural changes**	**Quantitative TEM**	**References**
HL-1 cells under starvation	Starvation ↑ autophagosomes and ↓ mitochondrial content	Mitochondria/cell sections ↓ from 35 to 11 after 3.5 h starvation, and 5 μM CsA correct it to 30; **Autophagosomes/cell section** **↑** **from 0.9 to 3.1 after starvation and CsA corrected it to 15**	Carreira et al., 2010
Adult cardiac progenitor cells in differentiation medium	Knockdown of the mitophagy receptors, *Bnip3* and *Fundc1* results in the presence of “donut”-shaped mitochondria containing electron-dense areas	No	Lampert et al., 2019
Drosophila muscle and heart	Knockdown of *Atg2, Atg9*, and *Atg18* resulted in elongated mitochondria. The TEM phenotypes were associated with cardiac hypertrophy and shortened life span	No	Xu et al., 2019
Hearts of the Parkin knockout (KO) mice	Smaller mitochondria, ↑Fis1 and ↓ dynamin-related protein 1 (Drp1), oxygen consumption normal in isolated mitochondria. In response to MI, more mitochondrial damage, and accumulation of autophagosomes in Parkin KO mouse hearts at the border zone	Mean mito area 0.5−>0.4 μm^2^	Kubli et al., 2013
Tamoxifen inducible Parkin KO (*via* myh6-MER-cre)	Heart mito in postnatal day 21 (P21) WT mice exhibited ovoid structure compared to P1. Parkin KO P21 mito did not differ from P1, but with abundant lipid droplets (LD)	No	Gong et al., 2015
Whole-body Parkin KO or cardiac (αMHC) Parkin overexpression (OE)	Using a polymerase γ (POLG) mutant mouse that develop cardiac hypertrophy, it was shown that neither Parkin KO or cardiac Parkin OE changed the POLG phenotype, whereas megamitochondria appear to be present in the POLG and the POLG:Parkin OE mice	No	Woodall et al., 2019
Dystrophin-deficient mice (mdx)	↓ Levels of LC3, P62, Pink1, and Parkin in the mitochondrial fraction; ↑ cristae-damaged mitochondria; ↓ the number of mitochondria in autophagosomes in response to 15 mg/kg DNP (a mitochondrial uncoupler)	At 3–4 months % mitochondria with loss of cristae 0.2−>1/4, at 12 months 1−>3.5 wt vs. mdx After injecting animals with DNP, % mitochondria with loss of cristae 1−> 8 wt vs. mdx; **% mitochondria in autophagosomes** 0.9−> 0.1 wt vs. mdx	Kang et al., 2018
AC16 cells with Dox	↑ Cristae-damaged mitochondria ↑ autophagosomes containing mitochondria	No	Yin et al., 2018
Rats with lentiviral pigment epithelial-derived factor (PEDF) followed by acute MI (AMI)	AMI ↓ the numbers of mitochondria and ↑ the numbers of mitophagosomes, PEDF further enhanced the change. In neonatal primary cardiomyocytes, PEDF increase cell survival in response to hypoxia, ↓ Parkin, and ↑ ULK1 and FUNDC1.	Number of **mitophagosomes** per unit area change from 0 to 2 by AMI, and to 4 if combined with PEDF. The numbers of mitochondria per unit area decreased from 20 to 5 by AMI, and further decreased by PEDF to 2	Li et al., 2018

*Quantification of autophagosomes and mitophagosomes are in bold*.

### Pink1 and Parkin and Mitophagy

Mitophagy is the specific autophagic degradation of mitochondrial cargo and is mediated by Pink1–Parkin-dependent and -independent pathways. TEM was used to examine cardiac ultrastructure in hearts of the Parkin KO mice under basal conditions and in the response to MI (Kubli et al., 2013). Parkin KO mice have smaller body weight (BW) and heart weight (HW), but the HW/BW is normal, so are the heart functions measured by echocardiography at 3–12 months of age. Disorganized and smaller mitochondria were present at baseline, with decreased Drp1 and increased Fis1 fission proteins, and normal oxygen consumption was measured in isolated mitochondria. In response to MI, WT mice subjected to MI had greater levels of Parkin protein, and Parkin KO mouse hearts had worsened functional outcome, a greater level of mitochondrial damage, and accumulation of autophagosomes at the border zone of the Parkin KO mice (Kubli et al., 2013) (Table 7). In mice with inducible Parkin deletion, heart mitochondria in KO mice at postnatal day 21 (P21) did not change compared to that at P1 (when tamoxifen was injected), whereas in WT mice they become ovoid (Gong et al., 2015). In this study, P21 Parkin KO mouse hearts also exhibited abundant LD (Gong et al., 2015). As mentioned above, studies in the whole-body Parkin KO mouse have shown larger diameter LD in the adult heart (Table 5) (Tong et al., 2019). The proof-reading defective mtDNA polymerase γ (POLG) mutant mouse strains that develop age-dependent cardiac hypertrophy are used as a model of aging, and it was shown using TEM that megamitochondria appear to be present in the POLG and the POLG:Parkin OE mice, whereas neither whole-body Parkin KO or cardiac Parkin OE rescued these POLG-dependent changes in mitochondrial morphology (Woodall et al., 2019).

**Figure 4:**
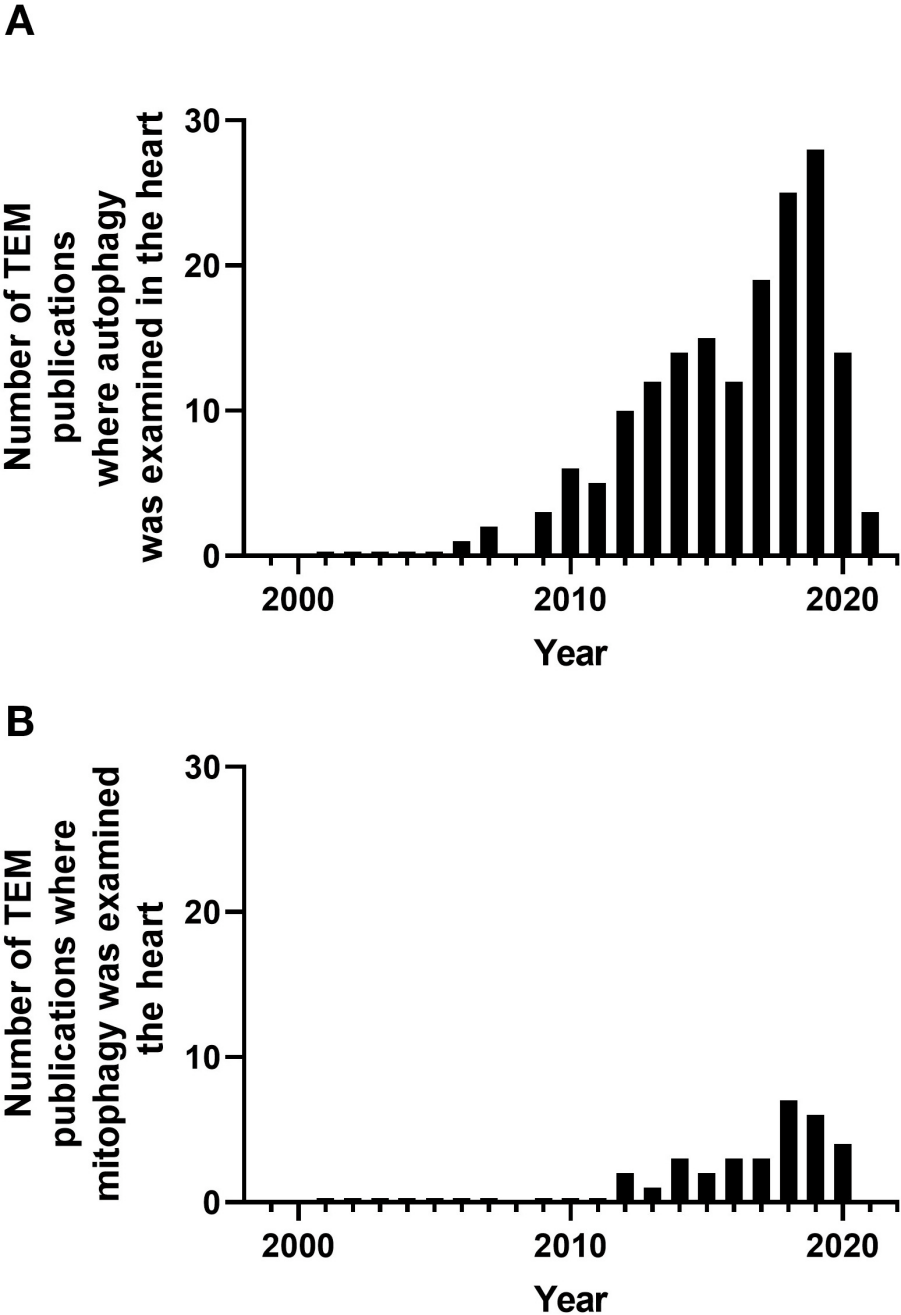
Number of peer-reviewed cardiovascular publications utilizing transmission electron microscopy to examine cardiovascular autophagy **(A)** and mitophagy **(B)**. Data taken from Pubmed analytics using a search of “Transmission electron microscopy,” “heart,” and “autophagy” or “mitophagy” for year 2000 to present.

In hearts from *mdx* mice [dystrophin-deficient mice as an animal model of Duchenne muscular dystrophy (DMD)], the percent of mitochondria with loss of cristae was increased both at 3–4 months of age and at 12 months of age compared to WT. With the exposure to mitochondrial uncoupler dinitrophenol, the percent of mitochondria in autophagosomes were lower in *mdx* mice compared to control mice shown using TEM. The levels of Pink1, Parkin, LC3II, and p62 in the mitochondrial fraction were also lower in *mdx* mice, consistent with decreased mitophagy (Kang et al., 2018). In AC16 cells, the Dox exposure resulted in the increased mitochondrial vacuolization with loss of cristae and the increase of autophagosomes containing mitochondria (Yin et al., 2018). In a study with rats, TEM was used to show that acute MI decreased the numbers of mitochondria and increased the numbers of mitophagosomes, and pigment epithelial-derived factor (PEDF) treatment further elevated the differences (Li et al., 2018). In neonatal primary cardiomyocytes, PEDF increased cell survival in response to hypoxia, decreased Parkin, and increased ULK1 and FUNDC1 (Li et al., 2018) (Table 7). Altogether, TEM studies on autophagy and mitophagy are generally supported with additional biochemical and cell biology studies, which delve into molecular cellular mechanisms and provide assessment of autophagy and mitophagy flux. Nonetheless, more and more studies are adopting TEM as one of the key elements to provide ultrastructural insights into these processes. Figure 4 illustrates the percentage of studies using TEM as a tool to examine autophagy and mitophagy in the heart.

### Mitochondrial Biogenesis, Fission, and Fusion

Many TEM studies that have investigated mitochondria often describe changes in the size and number of the mitochondria pool, which are regulated through the balance between biogenesis, mitochondrial degradation, and the dynamic nature of mitochondrial fission and fusion. Peroxisome proliferator-activated receptor gamma coactivator 1-alpha (PGC1) has been shown to play a key role in mitochondrial biogenesis. Adenoviral OE of PGC1? in rat neonatal cardiac myocytes resulted in enlarged mitochondria as viewed by TEM, which was associated with increased oxygen consumption (Lehman et al., 2000). Mice overexpressing cardiomyocyte PGC1α are Larger and develop DCM. One TEM study demonstrated numerous and enlarged mitochondria (Lehman et al., 2000). Despite changes in the mitochondrial function in PGC1α KO mice, there were no changes in mitochondrial morphology in the heart despite changes in the mitochondrial function (Arany et al., 2005). PGC1β KO mice showed normal heart function, whereas double KO (DKO) of PGC1α and PGC1β resulted in neonatal lethality with HF. TEM of the hearts from DKO mice showed decreased mitochondrial volume density and myofibril volume density at PD0.5, likely due to deficient mitochondrial biogenesis starting E17.5 (Lai et al., 2008). The heart and skeletal muscle PGC1α/β^−/−^ KO resulted in a decrease of the PGC1β mRNA level to 30% at birth in the heart (Martin et al., 2014). Mice start to die at 5 weeks of age with 14% survival rate at 20 weeks with progressive cardiomyopathy starting at 1 week of age. Mitochondria appear normal at postnatal day 1, but with fragmentation and elongation abnormality at 1 week, which progressively became worse and decreased mtDNA, mitochondrial proteins, and mitochondrial function were observed. Mitophagosomes were not observed, while genes involved in mitochondrial dynamics, including Mfn1, Mfn2, Opa1, Fis1, and Drp1 were decreased. Mfn1 exhibited the most dramatic decrease (Martin et al., 2014). TEM studies of mice with inducible PGC1β cardiac KO in a PGC1α^−/−^ background showed normal average mitochondrial size or volume density, while the numbers of mitochondria with cristae damage were increased and were associated with a decreased expression of CDP-diacylglycerol synthase 1, which catalyzes a key step in cardiolipin synthesis (Lai et al., 2014; Dorn et al., 2015). In Atgl-KO mice, the decrease in PGC1α and PGC1β has been proposed to mediate the observed mitochondrial defects, with increased mitochondrial diameter shown by TEM in the hearts of 10-week-old female mice (Haemmerle et al., 2011). More recently, a study generated moderate cardiac-specific PGC1α OE through knocking into ROSA26 locus in WT mice and in mice with a G3Terc^−/−^ (third generation of telomerase deficient; G3) background (Zhu et al., 2019). The G3 mice have decreased PGC1α levels and deficient basal and maximal mitochondrial function. TEM studies in 3-month-old WT, PGC1α OE, G3, and G3/ PGC1α OE mice demonstrated that PGC1α OE increased mitochondria numbers in both WT and G3 background (Zhu et al., 2019). Furthermore, at 12 months, PGC1α OE in WT background mice showed increased distorted mitochondria, and at 10 months, PGC1α OE in G3 mice showed decreased distorted mitochondria (Zhu et al., 2019). In addition to PGC1α, estrogen-related receptor α (ERRα) and ERRγ also have a combined role in mitochondrial biogenesis, as DKO of these genes resulted in lethal cardiomyopathy (Wang et al., 2015). At postnatal day 16, there was a significant decrease in the levels of mitochondrial electron transport chain proteins. TEM studies demonstrated distorted myofibrils, loss of clear boundaries between the A band and I band, fragmented mitochondria with loss of cristae, and decrease of mitochondrial size and perimeter (Wang et al., 2015). Postnatal KO of ERRα and ERRγ was generated using AAV9-cTnT-cre i.p. injection at postnatal day 0. At 5 weeks, there was an organelle containing vesicles suggestive of autophagic vacuoles, elongated or fragmented mitochondria, and LD in the DKO heart (Sakamoto et al., 2020). Taken together, these studies indicate that TEM has been a useful tool to demonstrate perturbation of mitochondrial quality control in the heart (Table 8).

**Table 8:** Mitochondrial quality control studies using TEM.

**The models**	**Ultrastructural changes**	**Quantitative TEM?**	**References**
Overexpression (OE) of PGC1α in cardiomyocytes	Adeno-PGC1α in rat neonatal cardiac myocyte resulted in enlarged mitochondria, with ↑ oxygen consumption; MHC-PGC1α mice are fatter, with dilated cardiomyopathy, and ↑ numerous and enlarged mitochondria	Mean total mitochondrial area/total cytoplasmic area in adeno-PGC1α cells was 57% higher relative to control (0.36 vs. 0.23)	Lehman et al., 2000
PGC1α whole body knockout (KO) at 3 months	No specific changes in hypertrophy, dilatation, or fibrosis; normal mitochondria volume despite changes in mitochondrial function	% Mitochondrial volume remained to be ~40%	Arany et al., 2005
PGC1β^−/−^ and double KO (DKO) of PGC1α and 1β	PGC1β^−/−^ appear normal, while DKO resulted in neonatal lethality with heart failure (HF). TEM of the E16.5, E17.5, PD0.5 hearts show lack of increase of heart size showed ↑ of mitochondria in WT, single KO, but not DKO hearts; ↓ mitochondrial volume density and normal myofibril volume density at PD0.5	Mitochondrial volume density from 0.3 to 0.1; myofibril volume density 0.35 μm^3^/μm^3^	Lai et al., 2008
Mck-cre mediated PGC1β KO with PGC1α-/–	30% levels of PGC1β mRNA at birth in the heart. Mice start to die at 5 weeks of age with 14% survival by 20 weeks. Progressive cardiomyopathy starting at 1 week of age. Mitochondria appear normal postnatal day 1, fragmentation and elongation abnormality at 1 week, and progressively worsen and also observed ↓ mtDNA, mito protein, and mito function. ↓ Mfn1, Mfn2, Opa1, Fis1 and Drp1, Mitophagosomes were not observed.	No	Lai et al., 2014
Tamoxifen inducible PGC1β cardiac KO with PGC1α-/–	1 month after tamoxifen, TEM shows normal average mitochondrial size or volume density, while ↑ the numbers of mitochondria with cristae damage, with ↓ expression of CDP-diacylglycerol synthase 1, which catalyzes a key step in cardiolipin synthesis	No	Lai et al., 2014; Dorn et al., 2015
Atgl-KO mice	↑ mitochondrial diameter, lipid droplet (LD) and glycogen granule numbers by TEM (10-week-old female), ↓ PGC1α and PGC1β, ↑ glucose/g tissue, ↓ mito size (by FACS), ↓ mtDNA (10-week-old female), impaired mitochondrial respiration (4- and 8-week-old male) in the hearts and led to cardiomyopathy	Mito diameter ~0.55 to ~0.62 μm (~18%) No quantification of LD or numbers of glycogen granules	Haemmerle et al., 2011
αMHC-PGC1α OE through knocking into the ROSA26 locus in both WT and G3Terc^−/−^ (third generation of telomerase deficient) background	The G3Terc^−/−^ mice have ↓ PGC1α levels and deficient mitochondrial function. PGC1α OE in wild-type (WT) mice led to cardiac dysfunction at 12 months and ↓ life span, and in G3 mice ↓ inflammatory cytokines at 10 months TEM studies in 3-month-old WT, PGC1α OE, G3, and G3/PGC1α OE mice demonstrated that PGC1α OE increased mitochondria numbers in both WT and G3 background, at 12 months PGC1α OE in WT mice ↑ distorted mitochondria, at 10 months PGC1α OE in G3 mice ↓ distorted mitochondria	Quantitation were performed at 3 months (but not 10 and 12 months): 10–12.5 mito number/view in WT vs. PGC1α OE 7.5–12 mito number/view in G3 vs. G3/PGC1α OE	Zhu et al., 2019
Estrogen-related receptor (ERR) α and ERRγ DKO with lethal cardiomyopathy	At P16 distorted myofibrils, loss of clear boundaries between the A band and I band, fragmented mitochondria with loss of cristae, ↓ mitochondrial proteins. TEM studies demonstrated ↓ of mitochondrial size and perimeter, some were wrapped by multiple double membranes	Mito Size (μm^2^): WT ~0.65, DKO ~0.2 Mito Perimeter (μm): ~4.2, DKO ~1.9	Wang et al., 2015
KO of ERRα and ERRγ using AAV9-cTnT-cre	At 5 weeks, there are vesicle engulfed organelles, elongated or fragmented mitochondria and LD in the DKO heart	No	Sakamoto et al., 2020

Mitochondrial fission proteins are comprised of Drp1 and mitochondrial fission 1 protein (Fis1), and the mitochondrial fusion proteins consist of the mitofusins 1/2 (Mfn1/2) and Optic atrophy protein 1 (Opa1), and alterations in the expression and activity of these proteins impact the available mitochondrial pool. Inducible cardiac-specific Drp1-KO resulted in DCM and HF at 6–8 weeks after KO. TEM demonstrated that mitochondria have preserved cristae; however, they were larger and more elongated (Song et al., 2015a). However, changes in mitochondrial autophagy and mitochondrial stress were confirmed using p62, LC3, LONP2, AFG3L2, and Hsp60 immunoblotting in this study (Song et al., 2015a). Furthermore, early cardiac-specific Drp1 KO mice were found to have compromised LV function at postnatal day 7 and decreased mitochondrial respiratory activity. TEM showed that myofibrils appeared unaffected; however, cardiomyocytes contained heterogeneous mitochondria and mitophagosome-like structures and mitochondrial vacuoles. Additional measurements of mitochondrial volume were made using 3D reconstruction with electron tomography (Kageyama et al., 2014), highlighting the need for additional technologies for the enhancement of traditional TEM. Similar findings were documented using the Drp1 inhibitor mdivi-1 (0.24–1.2 mg/kg; 15 min; i.v); however, the effects of mdivi were more pronounced in MI heart (Ong et al., 2010). Cardiac Parkin KO mice exhibited normal cardiac size, HW, and cardiac function for over 20 weeks. No cardiac OE in Parkin mice led to cardiac damage. TEM studies at 6 weeks after Parkin KO look normal, and Parkin transgenic mice at 30 weeks did not show abnormalities in mitochondrial content, mitochondrial area, and aspect ratio. Inducible Drp1 KO hearts exhibited loss of mitochondria 6 weeks after Drp1 deletion, whereas Parkin/Drp1 DKO delays cardiomyopathy of the Drp1 KO and partially restores mitochondrial content (Song et al., 2015b).

Optic atrophy 1 is involved in the fusion of the mitochondrial inner membrane, and its mutation causes autosomal dominant optic atrophy and the Behr syndrome in humans. Whole-body Opa1 heterozygous KO (+/–) mice exhibit cardiac dysfunction at 12 months but not at 3 months. However, TEM studies demonstrated disruption of orderly arrays of mitochondria between myofilaments and decreased mitochondrial density even at 3 months of age. Loss of cristae has been visualized at 12 months. Decreased complex IV activity and decreased mtDNA was seen at 3 months, and decreased complex I activity was seen at 12 months (Chen L. et al., 2012). A parallel study showed normal cardiac function of Opa^+/−^ mice at 6 months, whereas there was a decreased percentage of small mitochondria (<1 μm^3^ from 46 to 41%) and an increase in large mitochondria (>1.8 m^3^ from 21 to 27%) by MitoTracker image analyses. In these studies, mitochondria exhibited variability in size and shape at 3 months, with cristae deformation and the presence of dark material consistent with deficient fusion. There was also an increase of mean surface of individual mitochondrion, and there was no change in SR mitochondrial contacts (Chen L. et al., 2012; Piquereau et al., 2012).

The TEM imaging of cardiac-specific Mfn2 KO hearts has shown the increased presence of autophagosomes at 4 months, associated with increased LC3II and p62 protein levels, cardiac dysfunction at 17 months, and increased sensitivity to I/R injury at 6 months (Zhao et al., 2012). There was an increase of mitochondrial area; however, this neither correlated with changes in mitochondrial volume density nor in mtDNA (Zhao et al., 2012). Mfn2 deletion at early postnatal day 2 exhibited increased mitochondrial mean perimeter, decreased contact length with jSR, decreased Ca^2+^ uptake, and Ca^2+^-induced change of NAD(P)H/FAD+, whereas hearts from Mfn1 KO in the same study were normal (Chen Y. et al., 2012). However, in an independent study, where Mfn1 was deleted as early as embryonic day 9.5, mitochondria from Mfn1-KO mice were shown to have an increased presence of small, spherical, fragmented mitochondria; cardiac function and respiration in isolated mitochondria were maintained, but adult cardiac myocytes had better survival in response to H_2_O_2_ (Papanicolaou et al., 2012). Not surprisingly, as Drp1 deficiency resulted in cardiomyopathy and mitochondrial morphology changes in cardiac-specific Drp1-KO (Song et al., 2015a), significant changes in mitochondrial morphology and abnormal cardiac remodeling were also observed in the hearts of Mfn1/2 DKO mice (Song et al., 2015a). The cardiac expression of Mfn2 mutation (AA, T111A/S442A, Mfn2 that cannot be phosphorylated by PINK1) resulted in the presence of smaller and elongated mitochondria, associated with decreased succinate dehydrogenase (SDHB) complex II subunit compared to WT Mfn2 transgenics at 3 weeks of age (Gong et al., 2015). At 5 weeks of age, the Mfn2-AA mutant mice developed DCM (Gong et al., 2015). While Drp1 KO hearts have higher LV/wall thickness, Mfn DKO mice have enlarged hearts, but the LV/wall thickness remains unchanged. Mitochondrial area is decreased while the total content is increased in Mfn DKO (Song et al., 2015a). Using Mfn DKO, it was further shown that Mfn1/2 DKO mice are more resistant than WT mice to I/R (Hall et al., 2016). It was again shown that mitochondrial area decreased, while the presence of fragmented interfibrillar and cristae-disrupted mitochondria was reported, along with a decrease of maximal respiration in isolated mitochondria from isolated cardiomyocytes (Hall et al., 2016).

The analyses of TEM images using 3D electron tomography of the cardiomyocyte-specific Mfn2 KO mouse hearts suggest significant increases in mitochondrial volume and aspect ratio, which were associated with increased ER/SR–mitochondrial junctional distances and a reduction in the number of ER/SR–mitochondrial connections (Beikoghli Kalkhoran et al., 2017). As with the study using Mfn2 mutant mice *in vivo* (Gong et al., 2015), OE of Mfn2 (ad-Mfn2) in neonatal rat cardiomyocytes *in vitro* did not change mitochondrial number, length and size, aspect ratio, or number of mitophagic vacuoles (Xiong et al., 2019). However, addition of Ang II resulted in an accumulation of mitophagic vacuoles and increased mitochondrial size, indicative of increased mitochondrial damage (Xiong et al., 2019). Collectively, these studies clearly demonstrated that mitochondrial size and shape changes in response to genetic and pharmacological perturbations of fission and fusion proteins and in the context of heart diseases (Table 9).

**Table 9:** Additional mitochondrial fission and fusion studies in the heart using TEM.

**The models**	**Ultrastructural changes**	**Quantitative TEM?**	**References**
*i.v*. injection of mdivi-1 (1.2 mg/kg) 15 min	Mdivi-1 did not change mitochondrial morphology as assessed by TEM, while ↑ percent mitochondria that are >2 μm or 1 sarcomere in length, after 20 min regional ischemia compared to vehicle control	Percent mitochondria that are >2 μm or 1 sarcomere in length, 3.6% (vehicle) vs. 14.5% (with mdivi-1)	Ong et al., 2010
Drp (Myh6-cre) KO in the early postnatal days	Compromised LV function at postnatal day 7, decreased mitochondrial respiratory activity. Myofibrils appeared unaffected. Heterogeneous mito morphology in the Drp1KO heart, with branched tubules and large ovals. There are also vacuolar structures suggestive of mitophagosomes.	Tomography study with 3D reconstruction measurement demonstrated that Drp1 KO heart has: ↑ Mit volume by ~0.7 × 10^8^ nm^3^	Kageyama et al., 2014
Cardiac Drp1-KO (*via* myh6-MER-cre)	Dilated cardiomyopathy (DCM) and heart failure (HF) 6–8 weeks after KO. Mitochondria have preserved cristae; however, they were larger and more elongated. In this study, mitochondrial autophagy and mitochondrial stress were assessed using p62, LC3, LONP2, AFG3L2, and Hsp60 Western blots.	↑Mean area ctl 0.9, 3 weeks 1.8, 6 weeks 1.8 μm^2^ ↑Mito aspect ratio: clt 0.8, 3 weeks 1.7, 6 weeks 1.7 ↓Mito content: clt 48%, 3 weeks 35%, 6 weeks 30%	Song et al., 2015a
Parkin and Drp1 DKO mice (*via* myh6-MER-cre)	Parkin KO or overexpression (OE) did not result in cardiac dysfunction over 20 weeks. TEM at 6 weeks after Parkin KO look normal, Myh6-Parkin transgenic mice at 30 weeks did not show abnormalities in mito content, mito area and aspect ratio. TEM showed that Drp1 KO exhibit loss of mitochondria 6 weeks after Drp1 deletion, Parkin/Drp1 DKO delays cardiomyopathy of the Drp1 KO, and partially restore mitochondrial content	Mito content (% total cell): WT: 50%, Parkin KO: 50%, Drp1 KO: 30%, DKO: 42% Mito area (μm^2^): WT: 0.75 Parkin KO: 0.75 Drp1 KO: 1.3, DKO: 1.55 Mito aspect ratio: WT: 0.4 Parkin KO: 0.4 Drp1 KO: 0.7, DKO: 0.6	Song et al., 2015b
Whole-body Opa1 heterozygous knockout (KO) (+/–) mice	Cardiac dysfunction at 12 months but not 3 months ↓ Complex IV activity and ↓ mtDNA have been seen at 3 months, and ↓ complex I activity seen at 12 months. Disruption of orderly arrays of mitochondria between myofilaments, and ↓ mitochondrial density even at 3 months. Loss of cristae has been visualized at 12 months.	No	Chen L. et al., 2012
Whole-body Opa1 heterozygous KO(+/–) mice	At 6 months, no change of cardiac function, but ↓of small mitochondria (<1 μm^3^ from 46% to 41%) and increase of large mitochondria (>1.8 m^3^ from 21% to 27%) by MitoTracker image analyses. TEM showed that mitochondria exhibited variability in size and shape at 3 months, with cristae deformation and the presence of dark material consistent with deficient fusion. No change in SR-mitochondrial contacts	↑ Mean size of mitochondria: WT 0.5 μm^2^, +/– 0.7 μm^2^ ↑ mean surface of mitochondria % of a given surface: WT: 38%<0.4 μm^2^, 55% 0.4–0.8 μm^2^, 7%>0.8 μm^2^ +/–: 17%<0.4 μm^2^, 55% 0.4–0.8 μm^2^, 28%>0.8 μm^2^	Chen L. et al., 2012; Piquereau et al., 2012
Cardiac specific Mfn2 KO hearts (*via* Mlc2v-cre)	Cardiac dysfunction at 17 months, and increased sensitivity to I/R injury at 6 months. At 4 months, ↑ LC3II and p62 protein levels and ↑ autophagosomes by TEM, ↑ mitochondrial area, while no changes of mitochondrial volume density, nor of mtDNA.	↑ Mito area 0.65 μm^2^ WT, 1.05 μm^2^ in MFN2 CKO heart Mito volume density 0.4 μm^3^ /μm^3^ for both	Zhao et al., 2012
Mfn1 and Mfn2 KO (Myh6 “turbo” cre)	Mfn2 KO exhibit ↑ mito mean perimeter and area, ↓ contact length with jSR, ↓ Ca^2+^ uptake and Ca^2+^-induced change of NAD(P)H/FAD+, while Mfn1 KO is normal.	Ctl vs. Mfn2 ↑ Mean mito perimeter (μm) 3 vs. 3.9 ↑ Mean mito area (μm^2^) 0.68 vs. 1.1 ↓ Contact length with jSR (%) 48 vs. 34	Chen Y. et al., 2012
Using Myh6-cre to delete Mfn1 starting embryonic day 9.5	↑ Small, fragmented mitochondria; ↓ myofibril volume density; cardiac function and respiration in isolated mitochondria were maintained, but adult cardiac myocytes have better survival in response to H_2_O_2_	↓ Cross-sectional mito area 52–32 μm^2^ ↓ Mean diameter 3.05–2.36 μm ↓ Mean maximum diameter 1.12–0.7 μm ↑ numbers of 58–98/100 μm^2^ Normal mito volume density 42%, ↓ Myofibril volume density 44%−40%	Papanicolaou et al., 2012
Cardiac Mfn1/2 DKO (*via* myh6-MER-cre)	While Drp1 KO heart has ↑ LV/wall thickness, Mfn DKO mice have enlarged heart but the LV/wall thickness is unchanged. While Drp1 KO has larger mitochondria, Mfn DKO has smaller mitochondria	↓ Mito area ctl 0.75 μm^2^, 3 weeks 5 μm^2^, 6 weeks 0.4 μm^2^ Mito aspect ratio: 1.3 no change ↑ Mito content: clt 48%, 3 weeks 60%, 6 weeks 75%	Song et al., 2015a
Cardiac (Myh6) OE of Mfn2, Mfn2-AA	Mfn2-AA mice have smaller, abnormally shaped mitochondria in the heart starting at 3 weeks of age, associated with ↓ succinate dehydrogenase (SDHB) complex II subunit protein compared to wild-type (WT) Mfn2 transgenics, at 5 weeks of age, the Mfn2-AA mutant mice develop DCM	3 weeks: Mito content ↓ 46%−33%, Mito area ↓ 1.25–0.7 μm^2^, Mito aspect ratio ↑ 1.5–2.6	Gong et al., 2015
Cardiac Mfn1/2 DKO (*via* myh6-MER-cre)	Mfn DKO mice are more resistant to I/R injury, have smaller mitochondria, the presence of cristae-disrupted and fragmented interfibrillar mitochondria, maximal respiration ↓ in isolated mitochondria	↓ Mito area ctl 0.55–0.42 μm^2^ (5 weeks KO)	Hall et al., 2016
Mfn2 KO (cardiac- Myh6 “turbo” cre) 8–10 weeks	Extensive 3D analyses of mito volume, roundness, elongation, flatness, minimum mito-jSR distance	↑ Volume 0.6 vs. 0.85 μm^3^; ↓roundness 0.87 vs. 0.8; elongation similar at 0.40; ↑flatness 0.45 vs. 0.5; ↑ mito-jSR distance 12.5 vs. 17 nm; length of mito-jSR networks along Z-axis similar at 200 nm; ↓number of mito-jSR networks 18 vs. 9 nm	Beikoghli Kalkhoran et al., 2017
OE of Mfn2 (ad-Mfn2 vs. control of ad-ctl) in neonatal rat cardiomyocytes *in vitro*	No change of mitochondrial number, length, size, aspect ratio, or number of mitophagic vacuoles. However, addition of Ang II resulted in an accumulation of mitophagic vacuoles and increased mitochondrial size, indicative of increased mitochondrial damage	Mito autophagy (%) 0.05 ↑ by AngII to 0.12 in Ad-ctl, further ↑ 0.22 in Ad-Mfn2 by AngII, The number of mito/cell remains the same ~45 Aspect ratio 1.75 ↓ to 1.0 in Ad-ctl by AngII, to 1.25 in Ad-Mfn2 by AngII Av. mito area (μm^2^): ↓ 0.3–0.18 in Ad-ctl by Ang II, to 0.23 in Ad-Mfn2 by AngII Av. mito length (μm): ↓ 0.9–0.4 in Ad-ctl by AngII, to 0.6 in Ad-Mfn2 by AngII	Xiong et al., 2019

### Lipid Droplets

Transmission electron microscopy is capable of imaging LD at high resolution and has been used to describe conditions impacting the size and distribution of LD, such as cardiac lipotoxicity. In a study on Atgl, which is a lipase associated with LD homeostasis, TEM was used to examine cardiomyocyte LD size, glycogen granules, and mitochondrial diameters in Atgl-KO mice (Haemmerle et al., 2011). As discussed above in Table 8, Atgl-KO mice have lower PGC1α and 1β and reduced mitochondrial diameter and size, glycogen deposition, and LD deposition in the hearts of the KO mice, resulting in cardiomyopathy (Haemmerle et al., 2011). Additionally, cardiac OE of an Atgl-interacting protein, perilipin 5 (Plin5), resulted in the accumulation of LD in the vicinity of enlarged mitochondria in the heart, as shown by TEM (Wang et al., 2013). There was an increase in the thickness of LV wall while the cardiac function was normal and a significant alteration of gene expression occurred (Wang et al., 2013). Although the accumulation of LD may be related to, or affect, autophagy, these earlier studies did not investigate autophagy *per se*. In addition, there is a lack of studies on the use of TEM to study the role of lipophagy in heart diseases, and this warrants further investigation.

## Conclusions

When utilized appropriately, TEM is a powerful tool at not only visualizing the cardiac ultrastructure at a higher degree of resolution and magnification in comparison to traditional microscopy and imaging techniques but also at shedding significant light on the contribution of mitochondrial-derived signaling processes to dysregulation of the myocardium during health and disease. Despite the fact that TEM can be costly, sample preparation (and analyses) can be laborious and can introduce artifacts, these issues can be overcome with appropriate training. Importantly, TEM is a powerful imaging technique in the cardiovascular field and provides the powerful information regarding cardiac ultrastructure and signaling processes, which readily complements other imaging techniques. For example, for dynamic events, such as mitochondrial fission/fusion or engulfment of cargo by autophagosomes, live-cell imaging is preferred as TEM will only provide a snapshot of what is occurring with regard to mitophagy (and the number of mitophagosomes) but does not provide a real-time estimate of mitophagic flux. Furthermore, there is an additional level of complexity for assessing mitophagic flux, given the need for lysosomal inhibitors, which is currently not possible with traditional TEM imaging. Future advances in TEM sample preparation and staining and the development of highly innovative TEM imaging methodologies, such as 3D TEM, cryo-EM tomography, electron tomography, correlative light and electron microscopy (CLEM), and imaging systems, will likely advance the usage and capabilities of TEM in the cardiovascular field. The introduction of these new advances should help with the examination and quantification of measurements of 3D cardiac structures, such as mitochondria, so that accurate measurements may be obtained. In addition, newer labeling techniques, such as immunogold labeling, are promising new developments in the field of cardiac TEM. This allows specific proteins in tissues to be labeled during TEM, although this labeling method needs to be further developed since it is also associated with the production of image artifacts. Taken together, TEM provides essential information for visualization and quantification of cellular ultrastructure in both animal and human hearts. Furthermore, TEM has shed light on the presence of new cell types in the heart, and as technology improves, the capabilities of TEM will likely shift the field further.

## Author Contributions

HEC and JZ wrote the manuscript. MSK, SHL, VDU, MEY, and JCC critically read and edited the manuscript. All authors contributed to the article and approved the submitted version.

## Conflict of Interest

The authors declare that the research was conducted in the absence of any commercial or financial relationships that could be construed as a potential conflict of interest.

## Abbreviations

Atg9b: autophagy-related 9B
BW: body weight
CLEM: correlative light and electron microscopy
CVS: cardiovascular system
DCM: dilated cardiomyopathy
DKO: double knockout
Dox: doxorubicin
Drp1: dynamin-related protein 1
ECM: extracellular matrix
ER: endoplasmic reticulum
ERR: estrogen-related receptor α
HF: heart failure
HFD: high fat diet
HW: heart weight
I/R: ischemia/reperfusion
ICM: ischemic cardiomyopathy
IPSCs: induced pluripotent stem cells
jSR: junctional SR
KO: knockout
LD: lipid droplets
LV: left ventricular
LVAD: left ventricular assist device
MAM: mitochondrial-associated membrane
MDV: mitochondrial-derived vesicles
Mfn1: mitofusin 1
Mfn2: mitofusin 2
MI: myocardial infarction
Microtubule-associated protein 1A/1B-light chain 3: LC3
mito: mitochondria
mTOR: mechanistic target of rapamycin
Nrf1: nuclear respiratory factor 1
OE: overexpression
Opa1: optic atrophy 1
PBS: phosphate-buffered saline
PEDF: pigment epithelial-derived factor
PGC-1α: peroxisome proliferator-activated receptor gamma coactivator 1-alpha
Plin5: perilipin 5
SDHB: succinate dehydrogenase [ubiquinone] iron sulfur subunit, mitochondrial
SEM: scanning electron microscopy
sequestosome 1: p62
SR: sarcoplasmic reticulum
TAC: transverse aortic constriction
TEM: transmission electron microscopy.
